# A secondary analysis of autonomic function during high‐intensity interval exercise in adults with chronic stroke

**DOI:** 10.14814/phy2.70781

**Published:** 2026-02-16

**Authors:** Bria L. Bartsch, Saniya Waghmare, Mark Chertoff, Alicen A. Whitaker‐Hilbig, Sandra A. Billinger

**Affiliations:** ^1^ Department of Physical Therapy, Rehabilitation Science, and Athletic Training University of Kansas Medical Center Kansas City Kansas USA; ^2^ Department of Neurology University of Kansas Medical Center Kansas City Kansas USA; ^3^ Department of Physical Therapy Franklin Pierce University Manchester New Hampshire USA; ^4^ Department of Hearing and Speech University of Kansas Medical Center Kansas City Kansas USA; ^5^ Department of Anesthesiology Medical College of Wisconsin Milwaukee Wisconsin USA; ^6^ Cardiovascular Center Medical College of Wisconsin Milwaukee Wisconsin USA; ^7^ University of Kansas Alzheimer's Disease Research Center Fairway Kansas USA; ^8^ Department of Physical Medicine and Rehabilitation University of Kansas Medical Center Kansas City Kansas USA; ^9^ Department of Cell Biology and Physiology University of Kansas Medical Center Kansas City Kansas USA

**Keywords:** autonomic nervous system, baroreflex sensitivity, cerebrovascular accident, heart rate variability, spectral analysis

## Abstract

Autonomic dysfunction post‐stroke negatively affects the cardiovascular system's ability to regulate heart rate and blood pressure response to exercise. While high‐intensity interval exercise (HIIE) is prescribed for stroke recovery, limited knowledge exists regarding how HIIE impacts autonomic function post‐stroke. Characterize autonomic nervous system response to HIIE post‐stroke. Heart rate and blood pressure were recorded during a 10‐min recumbent stepper HIIE bout, cool‐down, immediately post‐exercise, and 30‐min post‐exercise. Fast Fourier Transformation was used to determine low and high frequency beat‐to‐beat blood pressure and heart rate power spectral density at all timepoints and baseline baroreflex sensitivity. We tested for differences in spectral data between timepoints using Friedman's test. The influence of arterial stiffness, age, and beta‐blocker use on autonomic function was explored using linear regression. Twenty‐seven participants completed HIIE. The autonomic nervous system response to HIIE was blunted with neither low nor high frequency blood pressure or heart rate variability exhibiting significant changes from baseline during exercise (*p* > 0.05). Baroreflex sensitivity was impaired, with age (*p* = 0.03), arterial stiffness (*p* < 0.01), and beta‐blocker use (p = 0.03) affecting sensitivity. Autonomic function is blunted during HIIE post‐stroke and likely attributed to impaired baroreflex sensitivity and arterial stiffness.

## 1 INTRODUCTION

Autonomic nervous system (ANS) dysfunction, characterized by impaired sympathetic and parasympathetic modulation, is associated with elevated resting and exercise blood pressure, an increased risk for cardiac events, reduced exercise capacity, and impaired heart rate recovery following activity (Brook, 2000; Fu & Levine, 2013; Mancia & Grassi, 2014; Pecanha et al., 2014; Rondon et al., 2006). Following stroke, ANS dysfunction is highly prevalent, affecting up to 76% of individuals, and is inversely associated with functional recovery (McLaren et al., 2005; Nayani et al., 2016; Xiong et al., 2012, 2018). Importantly, ANS dysfunction holds prognostic value for all‐cause morbidity and future cardiac events (Fang et al., 2020) and, when present during or after exercise, may increase the risk of acute cardiac complications (Smith et al., 2005; Thompson et al., 2007).

Exercise is a cornerstone of stroke rehabilitation (Billinger et al., 2014), yet limited insight exists into how the ANS responds during exercise in individuals with stroke. As ANS function can impact exercise tolerance and blood pressure response to exercise, understanding ANS function is important for clinicians in stroke recovery (Fisher & Vianna, 2025; Fonseca et al., 2022; Schwendinger et al., 2024). Specifically, the sympathetic and parasympathetic branches of the ANS work in a synergistic manner to facilitate the cardiovascular response necessary for adequate tissue perfusion and oxygen delivery during exercise (Fisher & Vianna, 2025). This collaborative response is termed sympathovagal balance (Goldberger, 1999).

Most studies in individuals with stroke to date have focused on recovery after submaximal exercise, and these findings suggest impaired ANS function, including reduced baroreflex sensitivity (BRS) and attenuated vagal tone post‐stroke (Fonseca et al., 2022; Francica et al., 2015). However, little is known about ANS response during exercise itself, and a comprehensive evaluation incorporating both blood pressure variability (BPV) and heart rate variability (HRV) is needed. ANS function can be noninvasively assessed through measures such as BPV and HRV, which reflect sympathetic and parasympathetic regulation, respectively (Akselrod et al., 1981; Julien, 2006; Pagani et al., 1986; Parati et al., 1995; Shaffer & Ginsberg, 2017; Waghmare et al., 2024). Low‐frequency (LF) BPV provides insight into baroreflex control of blood pressure and sympathetic activity, and high‐frequency (HF) BPV is influenced by respiratory‐driven blood pressure oscillations (Parati et al., 1995; Waghmare et al., 2024). LF HRV reflects baroreflex control of heart rate and sympathetic activity, and HF HRV reflects vagal tone, or the parasympathetic influence of the vagus nerve on heart rate and blood pressure (Parati et al., 1995; Waghmare et al., 2024).

High‐intensity interval training has emerged as a promising modality for stroke rehabilitation for improving gait speed and cardiorespiratory fitness (Boyne et al., 2023; Marzolini et al., 2023). Emerging evidence in healthy adults suggests that an acute bout of high‐intensity interval exercise (HIIE) significantly challenges the autonomic nervous system, eliciting acute changes in BPV and HRV with the repetitive fluctuations between sympathetic drive and parasympathetic recovery (Waghmare et al., 2024). However, the ANS response to HIIE in individuals with chronic stroke remains largely unexplored. Therefore, the purpose of this secondary analysis was to characterize ANS function in individuals with chronic stroke, with a specific focus on responses to HIIE. Our primary dependent variables were LF and HF BPV. Secondary variables included LF and HF HRV and sympathovagal balance. Independent variables included exercise condition (rest, HIIE, cool‐down, and recovery). We hypothesized: (1) LF BPV and HRV would increase during exercise compared to baseline, due to greater sympathetic activation during high‐intensity intervals than during active recovery, and (2) HF BPV and HRV would decrease during HIIE. As an exploratory aim, we examined the associations of resting BRS with arterial stiffness, beta‐blocker use, and age due to the known effects that these variables have on ANS function in other populations, such as adults with hypertension (Best et al., 2014; Borzuola et al., 2020; Eraky et al., 2024; Ganguli et al., 2014; Jayaraman et al., 2008; Monahan, 2007; Stratton et al., 2003).

## 2 METHODS

We performed a secondary analysis of data from the Blood Flow Response and Acute Interval Exercise study (BRAIN; NCT04673994 (12/2020‐02/2023)), where the primary outcome was the cerebrovascular response to HIIE in individuals with chronic stroke (Whitaker et al., 2023). The Institutional Review Board of the University of Kansas Medical Center approved study procedures, and data collection was performed in accordance with the Declaration of Helsinki. All participants provided written informed consent. In brief, participants were 6 months to 5 years postischemic or hemorrhagic stroke, sedentary (<150 min of moderate‐intensity exercise per week), and able to follow a 2‐step command. Additional details on inclusion and exclusion criteria in the primary study are reported elsewhere (Whitaker et al., 2023).

For data collection, participants completed two laboratory visits. At visit 1, the Total Body Recumbent Stepper submaximal exercise test was performed to predict peak power output (Watts_max_) (Bartsch et al., 2024; Whitaker et al., 2023; Wilson et al., 2017). The submaximal exercise test involves a progression through up to 4 stages of increasing workload until either (1) 85% of the participant's age‐predicted maximal heart rate is reached, (2) all stages of the test are completed, or (3) the participant requests to stop. At visit 2, arterial stiffness was assessed using carotid‐femoral pulse wave velocity (PWV, SphygmoCor, Itasca, IL) following approximately 20 min of supine rest. For PWV, standard guidelines were followed (Townsend et al., 2015), and a thigh cuff was placed on the right lower extremity, and a tonometer over the right carotid pulse. Aortic length was estimated, and approximately 10 cardiac cycles were recorded to determine PWV. Additional details on exercise testing and arterial stiffness methodology can be found in the primary publication (Whitaker et al., 2023).

At visit 2, participants were then instrumented with a 5‐lead electrocardiogram (ECG; Cardiocard, Nasiff Associates, Central Square, NY) for continuous heart rate acquisition, and a finger cuff (Finometer; Finapres Medical System, Amsterdam, Netherlands) for beat‐to‐beat blood pressure (Whitaker et al., 2023). With equipment on, participants completed a 5‐min seated baseline recording (BL), 10‐min recumbent stepper HIIE bout, 2‐min cool‐down, 5‐min seated rest recording immediately post‐exercise, and a 5‐min seated rest recording 30‐min post‐exercise. For HIIE, 1‐min of high‐intensity exercise (~70% Watts_max_) was interspersed with 1‐min of active recovery exercise (~10% Watts_max_). The 2‐min cool down was performed at 15 watts.

### 2.1 Data acquisition and analysis

Aligned with our previous work, data were sampled at 500 Hz using an analog‐to‐digit unit (NI‐USB‐6212, National Instruments) and custom‐written MATLAB code (v2017a, The MathWorks, Inc., Natick, MA) (Waghmare et al., 2024; Whitaker et al., 2023).

#### 2.1.1 Blood pressure and heart rate variability

Beat‐to‐beat blood pressure and heart rate data were resampled at 10 Hz and examined in the spectral domain using LF and HF power spectral density. In line with our previous work, a 0.04–0.15 Hz range was used for LF, and 0.15–0.40 Hz for HF (Waghmare et al., 2024). Data recordings were separated by time, detrended, and joined into the following periods to optimize data resolution (Waghmare et al., 2024): Rest, high‐intensity bouts, active recovery bouts, and cool down. Using the power spectral density (cPSD) package in MATLAB, Fast Fourier transform (FFT) analyses with 100‐s Hanning windows and 50% superposition were used to determine the beat‐to‐beat (BTB) BPV and HRV power spectral density in LF and HF domains. Absolute BTB BPV (mmHg^2^/Hz) and HRV (beats/min^2^/ Hz) power spectral density were determined as the sum of power across the LF and HF domains. The ratio of LF/HF was used to signify sympathovagal balance (Waghmare et al., 2024).

#### 2.1.2 Baroreflex sensitivity

Custom‐written MATLAB code was used to determine baroreflex sensitivity at baseline based on R‐R cardiac interval and systolic blood pressure. For R‐R interval, the distance between each ECG R spike in milliseconds was determined, and for BTB systolic blood pressure, the systolic peak of each waveform was identified. R‐R interval and systolic blood pressure were then downsampled to 10 Hz, linearly interpolated, and detrended. To determine the median gain across the LF spectrum, which signifies BRS, FFT analyses were performed with a 100‐s Hanning window and 50% superposition. Systolic blood pressure was used as the input signal, and R‐R interval, the output signal (Bagnall‐Hare et al., 2024). In line with previous work showing that a coherence criterion precludes the accurate assessment of BRS in cardiovascular populations, a coherence criterion was not applied to our BRS analysis in people with stroke, who often have existing cardiovascular disease (La Rovere et al., 2008; Pinna et al., 2002; Pinna & Maestri, 2002). As BRS becomes lower, signifying greater impairment, coherence nears zero, and as such, averaging the gain of the transfer function across the LF band improves BRS accuracy in populations with cardiovascular disease (La Rovere et al., 2008; Pinna et al., 2002; Pinna & Maestri, 2002).

### 2.2 Statistical analysis

Sample size was calculated a priori using G‐Power, based on preliminary data from the BRAIN study (NCT04673994) (Whitaker et al., 2023). As LF and HF BPV were our primary outcomes, effect sizes were derived from BRAIN study means and standard deviations. Data were not normally distributed and lacked homoscedasticity. Therefore, the Wilcoxon signed rank test option was applied in G‐Power. To achieve 80% power with *α* = 0.05 and effect sizes of 0.82 and 3.79 for LF and HF BPV during HIIE, respectively, 11 participants were required. The sample size used in this project aligns with our previous work exploring BPV and HRV in healthy young adults (Waghmare et al., 2024).

Normality was assessed using the Shapiro–Wilk test, and BPV and HRV data were not normally distributed. As such, data were analyzed using Friedman's test, with Wilcoxon's signed rank test and Holm's method for post hoc analysis. Due to the nonparametric nature of the data, results are presented as median (interquartile range), unless otherwise specified.

To explore factors which may contribute to autonomic dysfunction, multiple linear regression was used to determine the influence of age, beta‐blocker usage, and arterial stiffness on baseline BPV, HRV, and BRS. Variables within the model were checked for multicollinearity by calculating variance inflation factors. Alpha was set to 0.05, and for multiple linear regression models, the adjusted *R* ^2^ is reported. Statistical analyses were performed using R Studio, version 4.3.3 (Posit Team, 2023).

## 3 RESULTS

Twenty‐seven participant data sets were initially included. Demographics are presented in Table 1. On average, participants were 60.74 (11.54) years of age, 40.74% female, and 31.19 (16.6) months post‐stroke. For BPV and HRV, 6 and 5 datasets were excluded due to signal noise, resulting in 21 and 22 complete datasets analyzed for HIIE, respectively.

**TABLE 1:** Participant demographics.

	All
Age (years)	60.74 (11.54)
Female *n* (%)	11 (40.74%)
Race *n* (%)	
White/Caucasian	22 (81.48%)
Black/African American	5 (18.52%)
Asian	0 (0%)
Native American	1 (3.7%)
Ethnicity *n* (%)	
Hispanic	0 (0%)
Non‐Hispanic	27 (100%)
Body mass index (kg/m^2^)	30.56 (5.82)
Type of stroke, ischemic *n* (%)	21 (77.78%)
Stroke laterality, right *n* (%)	14 (51.85%)
Stroke location *n* (%)	
Cortical	19 (70.37%)
Subcortical	6 (22.22%)
Cerebellar	1 (3.70%)
Brainstem	1 (3.70%)
Time post‐stroke (months)	31.19 (16.6)
Modified Rankin Scale	2 (1–3)^a^
Hypertension *n* (%)	21 (77.78%)
Beta‐blocker medication *n* (%)	13 (48.15%)
Statin medication *n* (%)	24 (88.89%)
Pulse wave velocity (m/s)	9.55 (2.28)

*Note*: Mean (standard deviation) unless otherwise specified.

^a^ Median (interquartile range).

### 3.1 Baseline baroreflex sensitivity

Median gain across the LF spectrum, which signifies BRS, was 2.92 (3.18–6.33) with a coherence of 0.46 (0.41–0.57), suggesting reduced BRS when compared to the existing literature in healthy individuals and cardiovascular populations (Giannoni et al., 2022; Laitinen et al., 1998; Pinna et al., 2002; Radaelli et al., 2023). Within our model, age, beta‐blocker use, and arterial stiffness accounted for 40.71% of the variance observed in BRS (*p* < 0.01, *R* ^2^ = 0.41), with all three variables emerging as significant predictors (age: *p* = 0.03, *β* = −0.15; beta‐blocker use: *p* = 0.03, *β* = 3.49; arterial stiffness: *p* < 0.01, *β* = −0.94). This finding suggests that both increased age (Borzuola et al., 2020; Jayaraman et al., 2008; Monahan, 2007) and arterial stiffness (Best et al., 2014; Monahan, 2007; Stratton et al., 2003) negatively affect BRS, while beta‐blocker use enhances BRS (Eraky et al., 2024; Ganguli et al., 2014).

### 3.2 Beat‐to‐beat blood pressure variability

**FIGURE 1:**
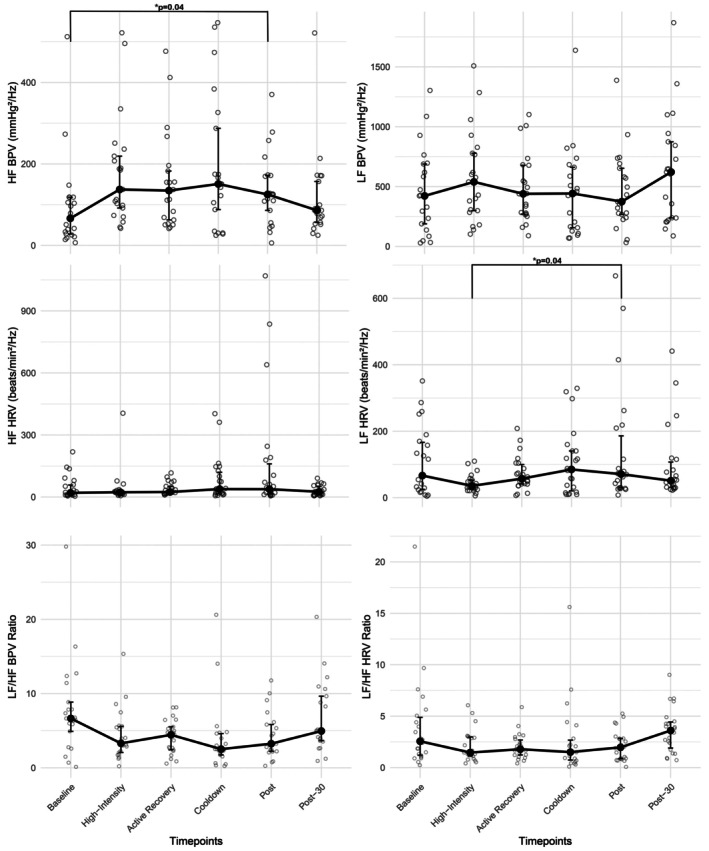
Blood pressure and heart rate variability. Cool down signifies the 2‐min cool‐down post‐exercise; Post, the 5‐min recording immediately post‐exercise; Post‐30, the 5‐min recording 30‐min post‐exercise; BPV, blood pressure variability; HF, high frequency; HRV, heart rate variability; Hz, hertz; LF, low frequency; min, minute; mmHg, millimeters of mercury.

Results for LF, HF, and LF/HF BPV are presented in Table 2 and Figure 1. LF BPV increased slightly during exercise, but no significant differences were present across timepoints (*p* = 0.23). Similarly, HF BPV exhibited a nonsignificant increase during exercise (High‐intensity, *p* = 0.13; Active recovery, *p* = 0.17). Immediately post‐exercise, however, HF BPV was significantly lower than baseline (*p* = 0.04). Friedman's test identified a significant overall effect across time for the LF/HF ratio (*p* = 0.02); however, subsequent post hoc comparisons did not detect significant differences between individual timepoints.

**TABLE 2:** BPV and HRV.

	Baseline	High‐intensity	Active recovery	Cooldown	Post	Post‐30	*p* Value
LF BPV mmHg^2^/Hz	421.93 (188.42–688.69)	539.06 (299.04–778.46)	439.38 (270.24–677.58)	442.06 (156.26–661.04)	374.24 (266.97–653.08)	621.13 (238.00–873.85)	*p* = 0.23
HF BPV mmHg^2^/Hz	66.41 (27.85–118.31)	137.32 (91.80–219.18)	134.57 (62.55–182.48)	150.78 (87.88–287.51)	125.11 (86.06–171.59)	86.12 (56.64–156.82)	*p* < 0.01*
LF/HF BPV	6.65 (4.91–8.85)	3.30 (2.05–5.58)	4.44 (2.42–5.52)	2.51 (1.73–4.61)	3.24 (2.21–5.82)	4.95 (3.63–9.64)	*p* = 0.02*
LF HRV (beats/min^2^)/Hz	66.44 (25.13–166.58)	35.60 (25.07–52.82)	57.24 (41.00–99.79)	84.94 (20.54–140.55)	70.90 (29.74–186.08)	51.14 (31.24–107.33)	*p* = 0.04*
HF HRV (beats/min^2^)/Hz	20.35 (10.58–58.04)	23.28 (15.77–26.63)	24.39 (18.86–50.52)	38.43 (18.09–119.78)	38.06 (14.38–160.46)	25.54 (10.62–50.59)	*p* = 0.46
LF/HF HRV	2.56 (1.21–4.89)	1.46 (1.13–2.97)	1.79 (1.23–2.68)	1.51 (0.73–2.67)	1.97 (0.86–2.83)	3.60 (1.90–4.43)	*p* = 0.25

*Note*: Median (interquartile range). *p* Value presented for Friedman's test across timepoints.

Abbreviations: BPV, blood pressure variability; HF, high frequency; HRV, heart rate variability; Hz, hertz; LF, low frequency; mmHg, millimeters of mercury; Post, immediately post‐exercise; Post‐30, 30‐min post‐exercise.

* *p* < 0.05.

Although individual models for LF and HF BPV were not significant, the model predicting resting LF/HF BPV was significant, explaining 31% of the variance (*p* = 0.04, *R* ^2^ = 0.31). Arterial stiffness emerged as a significant negative predictor of LF/HF BPV (*p* = 0.01, *β* = −2.01), suggesting that the shift in autonomic balance post‐stroke may occur with greater arterial stiffness (Al‐Wahab et al., 2024; Nakao et al., 2004; Nardone et al., 2020), even in the absence of strong associations with individual spectral components.

### 3.3 Beat‐to‐beat heart rate variability

Results for LF, HF, and LF/HF HRV are also presented in Table 2 and Figure 1. LF HRV decreased from baseline during exercise, but did not reach statistical significance. However, LF HRV during high‐intensity intervals was significantly lower than immediately post‐exercise (*p* = 0.04). For HF HRV, no significant differences were present across timepoints (*p* = 0.46). Similarly, no significant differences were present for LF/HF HRV (*p* = 0.25).

In the multiple linear regression models including age, beta‐blocker use, and arterial stiffness, the model did not reach statistical significance for LF HRV (*p* = 0.06, *R* ^2^ = 0.24) and HF HRV (*p* = 0.53, *R* ^2^ = 0.25). Although the overall model for LF was not significant, it accounted for 24.19% of the observed variance, with age emerging as a significant negative predictor (*p* = 0.02, *β* = −5.31). Similarly, for HF, the model explained 25.45% of the variance, and both age (*p* = 0.02, *β* = −2.87) and beta‐blocker use (*p* = 0.03, *β* = 56.50) were identified as significant predictors. In contrast, the model for LF/HF HRV was not significant (*p* = 0.13, *R* ^2^ = 0.15), suggesting limited explanatory value of these variables for sympathovagal balance in individuals post‐stroke.

## 4 DISCUSSION

This study provides new evidence on how the autonomic nervous system (ANS) responds to HIIE as well as post‐HIIE in individuals with chronic stroke, using LF and HF components of BPV and HRV. Our findings reveal a blunted autonomic response both during and after HIIE, suggesting impairments in sympathetic activity, vascular compliance, baroreflex sensitivity, and autonomic regulation. These results offer important new insights into post‐stroke physiology and highlight the broader impact of stroke‐related vascular and autonomic dysfunction on the body's ability to adapt to exercise.

### 4.1 Blood pressure and heart rate variability during HIIE

#### 4.1.1 Blood pressure variability

Compared to baseline, no significant changes were observed in LF or HF BPV during HIIE. The lack of a clear autonomic response during HIIE may reflect underlying impairments in vascular compliance, baroreflex sensitivity, or overall autonomic nervous system regulation post‐stroke. These findings differ from those observed in healthy, young adults completing an identical recumbent stepper HIIE bout (10 min in duration, 1‐min high‐intensity [70% Watts_max_], interspersed with 1‐min active recovery [10% Watts_max_]) where LF BPV increased significantly from baseline during active recovery, and HF BPV was significantly greater than baseline during both high‐intensity and active recovery intervals (Waghmare et al., 2024). In healthy individuals, this response can be expected as vascular compliance, vasomotor tone mediated by the vagus nerve, and BRS allow for the dynamic response of LF and HF BPV during exercise. Specifically, vascular compliance allows for changes in artery diameter in response to autonomic and mechanical signaling with exercise, vasomotor tone promotes increased parasympathetic activity during recovery periods, and BRS allows for the adjustment of heart rate in response to changes in blood pressure, given those changes are sensed by baroreceptors in the aortic arch and carotid arteries (Gourine & Ackland, 2019; Green et al., 2017; Green & Smith, 2018; Nobrega et al., 2014). As such, the differences between these findings in healthy individuals and participants with stroke suggest that post‐stroke pathophysiology may negatively impact autonomic response to HIIE. However, research regarding LF and HF BPV during exercise in stroke is nascent, highlighting the need for further research. Pathologic processes to consider due to the common cardiovascular etiology of stroke (Lui et al., 2025) include arterial stiffness and reduced BRS.

#### 4.1.2 Baroreflex sensitivity

Collectively, our participants demonstrated a median resting BRS gain value of 2.92 ms/mmHg. This value is well‐below BRS values observed in healthy individuals (6–19.5 ms/mmHg) (Laitinen et al., 1998; Pinna et al., 2002) and slightly below other cardiovascular populations (3–7 ms/mmHg) (Giannoni et al., 2022; Pinna et al., 2002; Radaelli et al., 2023), supporting the premise that impaired BRS may be contributing to the autonomic dysfunction observed within our participants with stroke. Additionally, we found that age, beta‐blocker use, and arterial stiffness were significant predictors of BRS, where age and arterial stiffness exhibited negative effects on BRS, and beta‐blocker use was protective due to the ability of these medications to shift the autonomic nervous system in favor of parasympathetic control (Eraky et al., 2024; Ganguli et al., 2014). Age has been well‐documented throughout the literature as having a negative effect on autonomic function, with the afferent and efferent limbs of BRS processing declining in function with age (Borzuola et al., 2020; Jayaraman et al., 2008; Monahan, 2007). Peripherally, the afferent limb is affected by increased arterial stiffness, which dampens the ability of baroreceptors to detect changes in blood pressure (Monahan, 2007). At the efferent level, vagal tone decreases with age, leading to either slower or blunted heart rate responses with exercise (Best et al., 2014; Monahan, 2007; Stratton et al., 2003). In our participants, we found an average PWV value of 9.55 m/s, which indicates increased arterial stiffness when compared to healthy individuals of similar age (Baldo et al., 2018; Diaz et al., 2018), and is common post‐stroke due to the high prevalence of cardiovascular disease risk factors in this population (Boehme et al., 2017). As such, stroke may further exacerbate the decline of BRS and autonomic function beyond that observed in healthy aging individuals. As such, stroke may further exacerbate the decline of BRS and autonomic function beyond that observed in healthy aging individuals. While assessing BRS during exercise may have provided additional information on ANS function post‐stroke, the total duration of concatenated high‐intensity and active recovery exercise present in our recording likely precludes an accurate assessment of BRS during HIIT. Typically, at least 5 min of steady‐state exercise is used for BRS recordings during exercise (Fisher et al., 2009). Further research is required to better understand the underlying mechanisms of autonomic dysfunction post‐stroke.

#### 4.1.3 Heart rate variability

For HRV, our participants with stroke demonstrated no significant change in LF or HF from baseline to exercise. These HRV findings also contrast those observed in healthy, young adults, where a significant decrease in LF from baseline was observed during HIIE, and LF HRV during the high‐intensity intervals was significantly lower than during active recovery intervals (Waghmare et al., 2024). Here, the decrease in LF was likely due to reduced HRV, which is commonly observed with parasympathetic withdrawal during exercise (Michael et al., 2017). Indeed, in healthy participants, HF was significantly lower than baseline during high‐intensity intervals (Waghmare et al., 2024). As such, these contrasting findings between our participants with stroke and healthy individuals further reinforce the premise that impaired BRS and autonomic dysfunction are present post‐stroke, negatively affecting heart rate and blood pressure response to HIIE. Interestingly, another study exploring LF and HF HRV response to moderate‐intensity treadmill‐based exercise in chronic stroke found that LF HRV increased during exercise, and HF HRV was reduced (Raimundo et al., 2013). While this pattern also suggests that autonomic dysfunction may be present during exercise post‐stroke, this HRV response is different than what we observed in our current study. This is likely due to differences in the exercise protocols, such as exercise modality, intensity, and duration. For example, treadmill‐based exercise utilizes more muscle mass than seated exercise, and greater muscle mass involvement leads to greater activation of sympathetic activity through the exercise pressor reflex (Murphy et al., 2011). Additionally, a continuous moderate‐intensity protocol may not challenge the autonomic system in the same oscillatory manner as HIIE, leading to varied responses in autonomic function. Further considerations include potential differences in participant demographics, such as medication use, stroke chronicity, and age.

### 4.2 Blood pressure and heart rate variability following HIIE

No significant differences were observed for LF BPV following HIIE. However, HF BPV immediately post‐exercise was significantly greater than at baseline. This was likely facilitated by an elevated respiratory rate and depth during the initial recovery period and mirrored the HF BPV response observed in healthy individuals completing an identical HIIE bout (Waghmare et al., 2024). Specifically, following exercise, respiration remains elevated to promote the removal of carbon dioxide and lactic acid from the body (Børsheim & Bahr, 2003). These findings in HF BPV are similar to those observed in a trial where researchers explored BPV for 20‐min following a submaximal exercise test in chronic stroke (Francica et al., 2015). However, in this trial, researchers also found LF BPV to be significantly elevated following submaximal exercise. The discrepancy between findings in LF BPV between our study and the latter may be due to differing exercise intensities, as well as differing participant characteristics. Within our trial, 48% of participants were on beta‐blockers, which promote parasympathetic control (Eraky et al., 2024; Ganguli et al., 2014). In the latter study, beta‐blocker use was exclusionary and may explain the elevated sympathetic activity post‐exercise.

For LF and HF HRV post‐stroke, no significant differences were observed between post‐exercise timepoints, which is in contrast of the findings observed in healthy adults where LF and HF HRV during cool‐down were significantly lower than at baseline, and HF HRV remained lower immediately post‐exercise (Waghmare et al., 2024). However, these discrepancies do not necessarily suggest autonomic pathology during the recovery phase in participants with stroke. Given that healthy individuals demonstrated greater changes from baseline in LF and HF HRV during HIIE (Waghmare et al., 2024), the healthy participants likely required increased time for autonomic nervous system recovery. Interestingly, one study did explore the impact of mixed circuit training on LF and HF HRV post‐exercise in persons with chronic stroke (Fonseca et al., 2022). At 40‐min post‐exercise, LF HRV was significantly higher than at baseline, and HF, significantly lower (Fonseca et al., 2022). The differences in findings between this study and ours are likely due to the use of varying exercise modalities, where the resistance exercises involved in mixed circuit training led to greater sympathetic activation and parasympathetic withdrawal. Additionally, stroke severity and time post‐stroke can affect autonomic function (Cha et al., 2023; Liu et al., 2017; Wang et al., 2022), and participants within the mixed circuit training study were on average 91 months post‐stroke (Fonseca et al., 2022) versus 31 months post‐stroke in the present study (Fonseca et al., 2022).

### 4.3 Clinical relevance

Exercise is prescribed for stroke recovery (Billinger et al., 2014), with HIIE becoming a more commonly used method due to HIIE's ability to improve gait speed and endurance (Boyne et al., 2023; Gjellesvik et al., 2021). Thus, understanding how autonomic dysfunction may impact heart rate and blood pressure response to HIIE is important for clinicians to ensure patient safety. Clinicians should consider the effects of autonomic dysfunction on post‐exercise hypotension following HIIE. Following exercise cessation, cardiac output decreases, and sympathetic tone must increase to oppose exercise‐induced vasodilation (Michael et al., 2017; Thomas & Segal, 2004). In individuals with stroke, impaired BRS and autonomic dysfunction can lead to an impaired ability of the autonomic system to combat the decrease in cardiac output, resulting in post‐exercise hypotension (Fonseca et al., 2022; Lapointe et al., 2022; Low et al., 2012). In chronic stroke, research found that systolic blood pressure was reduced for 10 h following mixed circuit training (Fonseca et al., 2022). Given that increased exercise intensity is associated with greater and longer post‐exercise hypotension (Lu et al., 2024), monitoring blood pressure and heart rate following HIIE is important for clinicians prescribing exercise post‐stroke to decrease the risk of an adverse event. Further, autonomic dysregulation may exacerbate exercise intolerance, where a blunted autonomic response to exercise can lead to inadequate oxygen delivery to the brain and active musculature (Schwendinger et al., 2024). By understanding how autonomic dysfunction affects exercise performance and recovery, clinicians can optimize stroke recovery while ensuring patient safety.

Additionally, an important consideration is that while HIIE may acutely challenge the ANS in individuals with stroke, chronic engagement in HIIE may confer long‐term physiologic benefits. A recent review in individuals with and without cardiometabolic risk factors across the lifespan found that 2–12 weeks of HIIE led to improved autonomic modulation and vagal activity (Coretti et al., 2025). While this has not yet been explored in stroke, existing evidence supports the notion that chronic HIIE engagement may improve post‐stroke ANS function. For example, a systematic review of eight studies in individuals with stroke found that HIIE increases brain‐derived neurotrophic factor and vascular endothelial growth factor (Montero‐Almagro et al., 2024). Collectively, these neurotrophic factors promote angiogenesis and nitric oxide production (Biojone et al., 2015; Kermani & Hempstead, 2007; Lee et al., 2025), which may in turn improve vascular function and promote positive adaptations in autonomic function (Amiya et al., 2014). Future research is required, however, to explore the potential benefits of long‐term HIIE on post‐stroke ANS function.

### 4.4 Limitations

Although our study was powered to assess LF and HF BPV during HIIE, we recognize that stroke is a heterogeneous condition. Future studies may use our findings to inform future power analyses and further expand knowledge on ANS function during exercise post‐stroke. Although our study didn't include magnetic resonance imaging, future work should consider the influence of stroke location on BPV and HRV responses during exercise since stroke lesion location may impact ANS function during resting conditions (Chen et al., 2013; Tokgozoglu et al., 1999). Additionally, while we report baseline BRS and speculate on its potential impact on ANS responses during HIIE, direct investigation of BRS during HIIE could provide valuable insight into the interaction between BRS and ANS function. Future research should also consider the role of respiration, as HF BPV is influenced by respiratory‐driven oscillations in blood pressure (Parati et al., 1995; Waghmare et al., 2024), and HF HRV may be confounded by respiratory sinus arrhythmias which can occur with changes in respiratory rate during exercise (Hatfield et al., 1998; Strano et al., 1998). Given that respiratory changes are inherent to HIIE, the HF derived outcomes in this study should be interpreted with caution and guide future research. A systems‐based approach examining how the integration of these physiological processes influence ANS responses to exercise post‐stroke may provide valuable insight into how stroke alters cardiorespiratory and autonomic regulation, as individuals post‐stroke demonstrate impaired peak and reserve ventilation and cardiac output (Tomczak et al., 2008). Finally, a comparator group was not included in this project. As such, the potential presence of ANS impairment post‐stroke, as reviewed in the discussion and compared to findings in healthy individuals, should be interpreted with caution and examined in future research.

## 5 SUMMARY

In this study, we found that individuals with chronic stroke exhibit impaired baroreflex sensitivity at rest and a blunted autonomic response during and following high‐intensity interval exercise (HIIE). Specifically, expected parasympathetic withdrawal and sympathetic activation during exercise were diminished, and autonomic recovery post‐exercise was limited. These findings suggest that stroke‐related vascular and autonomic dysfunction alters the normal regulation of cardiovascular responses to exercise. Understanding these impairments is critical for optimizing exercise prescriptions and ensuring cardiovascular safety in stroke rehabilitation. Future research is needed to further elucidate the mechanisms underlying autonomic dysfunction during exercise and to identify strategies to improve autonomic regulation after stroke.

## AUTHOR CONTRIBUTIONS

BLB, SW, AAW, and SAB contributed to conceptualization. BLB, SW, AAW, and SAB contributed to data curation. BLB, SW, MC, AAW, and SAB were involved in methodology. BLB, SW, MC, and SAB performed formal analyses. BLB, SAB, and AAW participated in funding acquisition. All authors were involved in investigation and writing. SAB oversaw project administration and supervision.

## FUNDING INFORMATION

S.A.B. was supported in part by P30 AG072973. B.L.B. was supported in part by T32HD057850. REDCap at the University of Kansas Medical Center was supported by the National Center for Research Resources UL1TR002366.

## CONFLICT OF INTEREST STATEMENT

The authors declare no competing interests.

## ETHICS STATEMENT

This study was granted ethical approval by the University of Kansas Medical Center's Human Subjects Committee and Institutional Review Board. Prior to obtaining informed written consent, we provided verbal and written explanation of the experimental protocol, potential benefits and associated risks to all participants.

## Data Availability

Data are available from the corresponding author upon resonable request.
